# Safety and Metabolic Outcomes of Three-Port Laparoscopic Sleeve Gastrectomy Without Liver Retractor: A 2000-Patient Retrospective Study

**DOI:** 10.3390/medicina62061118

**Published:** 2026-06-08

**Authors:** Muzaffer Önder Öner, Fırat Aslan, Serhat Binici, Burhan Beger, Orhan Beger

**Affiliations:** 1 General Surgery Department, Faculty of Medicine, Nişantaşı University, 34398 Istanbul, Türkiye; 2 General Surgery Department, Van Yüzüncü Yıl University, 65090 Van, Türkiye; dr.aslan.2609@hotmail.com (F.A.); drserhatbinici@gmail.com (S.B.); 3 Pediatric Surgery Department, Van Yüzüncü Yıl University, 65090 Van, Türkiye; burhanbeger@hotmail.com; 4 Department of Anatomy, Faculty of Medicine, Gaziantep University, 27410 Gaziantep, Türkiye; obeger@gmail.com

**Keywords:** laparoscopic sleeve gastrectomy, bariatric surgery techniques, hepatic circulation preservation, obesity-related metabolic improvements

## Abstract

*Background and Objectives*: Laparoscopic sleeve gastrectomy (LSG) is one of the most commonly performed metabolic bariatric surgery procedures worldwide. However, conventional LSG generally requires liver retraction for adequate visualization of the operative field. This study aimed to evaluate the feasibility, perioperative safety, and metabolic outcomes of a modified three-port LSG technique performed without the use of a liver retractor. *Materials and Methods*: This retrospective single-center cohort study included 2000 consecutive individuals with obesity who underwent three-port laparoscopic sleeve gastrectomy between January 2020 and December 2023. All procedures were performed without mechanical liver retraction by two experienced bariatric surgeons. Operative outcomes, postoperative complications, weight loss parameters, metabolic variables, and histopathological findings were evaluated during a 12-month follow-up period. All included patients completed the predefined follow-up schedule. Postoperative complications were classified according to the Clavien–Dindo classification system. *Results:* The mean operative time, defined as skin-to-skin duration, was 30 ± 15 min, and the median hospital stay was 2.3 days. No conversion to open surgery, additional trocar placement, or rescue liver retractor use was required. The overall complication rate was 9.4%, with most complications classified as Clavien–Dindo grade I–II. Reoperation was required in three patients (0.15%), and no mortality was observed. Significant metabolic improvements were detected following surgery. Mean HbA1c levels decreased from 7.23% preoperatively to 5.67% at 12 months (*p* < 0.001), while BMI decreased from 42.6 kg/m^2^ to 28.7 kg/m^2^ (*p* < 0.001). Excess weight loss and total weight loss at 12 months reached 82.4% and 34.2%, respectively. Diabetes remission was achieved in 65.4% of patients with baseline type 2 diabetes mellitus. Continuous glucose monitoring findings demonstrated reduced postoperative glycemic variability. *Conclusions:* Three-port laparoscopic sleeve gastrectomy performed without a liver retractor appears to be a feasible and effective surgical approach when performed by experienced bariatric surgeons. The technique was associated with acceptable perioperative safety and favorable metabolic outcomes. However, because of the retrospective single-center design and absence of a conventional comparison group, definitive conclusions regarding superiority or equivalence to standard techniques cannot be established. Prospective multicenter comparative studies are required to validate these findings.

## 1. Introduction

Obesity is a major global health problem and is recognized as one of the leading contributors to metabolic disorders, cardiovascular diseases, and increased mortality. Metabolic bariatric surgery (MBS) has emerged as the most effective long-term treatment option for achieving sustained weight loss and improving obesity-related comorbidities. Among bariatric procedures, laparoscopic sleeve gastrectomy (LSG) has gained widespread acceptance due to its relative technical simplicity, favorable safety profile, and significant metabolic benefits [1,2].

Beyond its reduction in gastric reservoir volume, LSG exerts substantial metabolic effects. Previous studies have demonstrated that LSG improves insulin resistance, enhances glycemic control, and leads to remission of type 2 diabetes mellitus through mechanisms such as reduction in visceral adiposity and modulation of gut hormones [1,3]. Additionally, LSG has been associated with significant reductions in hepatic and pancreatic fat content, further contributing to improved metabolic outcomes [1,2,4]. These metabolic improvements are accompanied by favorable changes in lipid profiles, including increased high-density lipoprotein (HDL) levels and decreased triglyceride levels [3].

Despite these well-established benefits, technical aspects of LSG continue to evolve in order to optimize surgical outcomes and minimize perioperative risks. In standard LSG procedures, the use of a liver retractor is generally considered essential for adequate exposure of the operative field, particularly visualization of the gastric fundus and angle of His. However, liver retraction may be associated with certain drawbacks, including potential impairment of hepatic circulation, transient liver injury, and increased technical complexity during surgery. Moreover, the need for an additional port for liver retraction may reduce the minimally invasive nature of the procedure and negatively affect cosmetic outcomes. Previous studies evaluating complications and technical aspects of minimally invasive upper gastrointestinal surgery have also suggested that prolonged mechanical liver retraction may contribute to transient hepatic injury and technical difficulties, particularly in patients with obesity-related hepatosteatosis [5,6].

Recent advances in minimally invasive surgery have focused on reducing the number of ports and minimizing the use of auxiliary instruments. Reduced-port and single-incision techniques have been shown to decrease postoperative pain, shorten recovery time, and improve patient satisfaction [7,8]. However, concerns remain regarding the adequacy of surgical exposure and the safety of these modified approaches. In particular, adequate visualization may become technically challenging in patients with hepatomegaly, hepatosteatosis, or obesity class III. Furthermore, although several studies have investigated reduced-port LSG techniques, data regarding the feasibility and outcomes of LSG performed without the use of a liver retractor remain limited.

In addition to technical considerations, LSG is associated with certain postoperative challenges, including gastroesophageal reflux and variability in long-term weight maintenance [9,10,11,12]. These issues highlight the need for continuous refinement of surgical techniques to improve both short- and long-term outcomes. In particular, modifications that preserve surgical safety while enhancing recovery and minimizing invasiveness are of significant clinical interest.

Therefore, there is a clear need for large-scale studies evaluating modified LSG techniques that eliminate the use of liver retractors while maintaining surgical efficacy and safety. The present study aims to evaluate the safety, feasibility, and metabolic outcomes of a modified three-port laparoscopic sleeve gastrectomy technique performed without the use of a liver retractor.

We hypothesized that the three-port liver retractor-free LSG technique would be feasible and associated with acceptable perioperative and metabolic outcomes in individuals with obesity when performed by experienced bariatric surgeons.

## 2. Materials and Methods

### 2.1. Ethics Statement

This retrospective, single-center cohort study was conducted in accordance with the Declaration of Helsinki and approved by the Health Sciences Research Ethics Committee (approval date: 2 December 2024; approval number: E-97429853-050.04-). Due to the retrospective design of the study, the requirement for informed consent was waived. The study was reported in accordance with the Strengthening the Reporting of Observational Studies in Epidemiology (STROBE) guidelines.

### 2.2. Study Design and Patient Selection

A total of 2000 consecutive individuals with obesity who underwent laparoscopic sleeve gastrectomy (LSG) between January 2020 and December 2023 at İzmir Ekonomi University Medical Point Hospital were included in the study. Patients aged between 18 and 65 years with a body mass index (BMI) ≥ 40 kg/m^2^ or ≥ 35 kg/m^2^ in the presence of at least one obesity-related associated medical problem were eligible. Patients with known malignancy, severe hepatic insufficiency, prior bariatric surgery, or contraindications to general anesthesia were excluded. All patients were evaluated preoperatively by a multidisciplinary team including endocrinology, cardiology, pulmonology, and psychiatry specialists.

Only patients who completed scheduled postoperative follow-up evaluations at postoperative day 15 and months 1, 3, 6, and 12 were included in the final analysis. Therefore, no loss to follow-up was present within the analyzed cohort.

### 2.3. Surgical Technique

**Figure 1:**
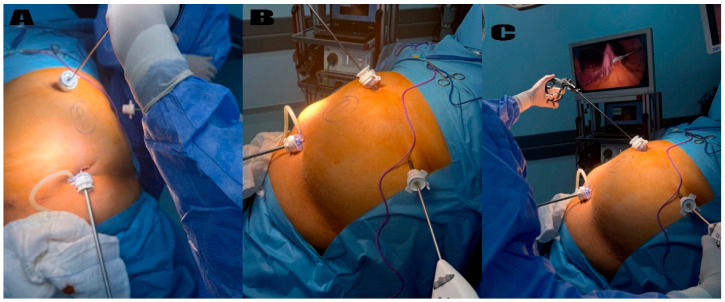
Surgical steps of the modified three-port laparoscopic sleeve gastrectomy technique performed without liver retraction (**A**–**C**).

All procedures were performed using a standardized modified three-port laparoscopic sleeve gastrectomy technique without the use of a liver retractor. Under general anesthesia, patients were placed in the reverse Trendelenburg position. A 12-mm camera port was inserted in the supraumbilical midline, a 5-mm working port was placed in the left upper quadrant, and a 12-mm working port was positioned approximately 5 cm inferior to the xiphoid process (Figure 1).

Instead of using a mechanical liver retractor, operative exposure was achieved through reverse Trendelenburg positioning, left-sided table tilt, controlled traction of the gastric fundus, and upward tension applied along the greater curvature during dissection. Elevation of the stomach and omentum facilitated gravitational displacement of the left hepatic lobe, allowing adequate visualization of the angle of His and left diaphragmatic crus without additional liver retraction. Intermittent external abdominal wall compression was used when necessary to optimize exposure in patients with increased visceral adiposity or hepatosteatosis. No additional trocar placement, conversion to open surgery, or intraoperative liver retractor use was required in any patient. All procedures were performed by two experienced bariatric surgeons.

Gastric mobilization was performed along the greater curvature, starting 2–4 cm proximal to the pylorus and extending to the angle of His. A 38F (Medsil, Moscow, Russia) bougie was used for calibration during sleeve creation. Gastric transection was carried out using a linear stapler with a tristapler purple cartridge, and no staple line reinforcement or oversewing was applied. Intraoperative leak testing was performed using methylene blue (Biofarma, Istanbul, Türkiye), and no routine drain placement was performed.

Operative time was defined as skin-to-skin duration, beginning with the initial skin incision and ending with completion of skin closure.

### 2.4. Postoperative Care and Follow-Up

Postoperatively, patients were mobilized early, and oral intake was initiated on the first postoperative day. Discharge criteria included adequate oral intake, stable vital signs, and satisfactory clinical condition. Patients were followed at regular intervals, including postoperative day 15 and months 1, 3, 6, and 12. Clinical and metabolic parameters were recorded during follow-up visits.

### 2.5. Data Collection and Outcome Measures

Demographic data, obesity-associated medical problems, operative findings, and postoperative outcomes were obtained from hospital records. Primary outcomes included operative time, length of hospital stay, and early postoperative complications. Postoperative complications were classified according to the Clavien–Dindo classification system. Secondary outcomes included weight loss parameters such as percentage of excess weight loss (%EWL) and total weight loss (%TWL); glycemic control parameters including HbA1c, fasting glucose, and HOMA-IR, diabetes remission rates; and changes in lipid profile. Diabetes remission was defined as HbA1c < 6.5% in the absence of antidiabetic medication use.

Laboratory parameters, including HbA1c, fasting glucose, lipid profile, and HOMA-IR, were recorded preoperatively and at 1 and 12 months postoperatively. In a subgroup of patients undergoing postoperative metabolic assessment, continuous glucose monitoring (CGM) data obtained from routinely used clinical monitoring systems were retrospectively reviewed to evaluate 24-h glycemic variability patterns before and after surgery. Due to the retrospective design and variability in monitoring protocols, CGM findings were evaluated descriptively.

### 2.6. Statistical Analysis

Statistical analyses were performed using IBM SPSS Statistics version 26.0 (IBM Corp., Armonk, NY, USA). Continuous variables were expressed as mean ± standard deviation or median with interquartile range, depending on distribution, and categorical variables were presented as number and percentage. Normality of distribution was assessed using the Shapiro–Wilk test. Comparisons of repeated measurements were performed using repeated measures ANOVA or the Friedman test where appropriate, with Bonferroni correction applied for multiple comparisons. Categorical variables were compared using the Chi-square test or Fisher’s exact test. A *p*-value of less than 0.05 was considered statistically significant.

Because all eligible patients with complete follow-up during the study period were included, no formal sample size calculation was performed. Missing data analysis was not required, as only patients with complete follow-up datasets were included in the final analysis. Additionally, 95% confidence intervals (95% CIs) were calculated for major perioperative and key metabolic outcomes.

## 3. Results

A total of 2000 consecutive individuals with obesity who underwent three-port laparoscopic sleeve gastrectomy without liver retraction between January 2020 and December 2023 were included in the final analysis. All included patients completed the predefined postoperative follow-up schedule at postoperative day 15 and months 1, 3, 6, and 12. No conversion to open surgery, additional trocar placement, or intraoperative liver retractor use was required.

The mean age was 38.7 years (range: 22–65), and the majority of patients were female (71.2%). The mean preoperative body mass index (BMI) was 42.6 kg/m^2^ (range: 35–55), corresponding predominantly to obesity class III. The most common associated medical problems were hypertension (25.9%) and type 2 diabetes mellitus (23.2%), followed by sleep apnea (18.9%) and dyslipidemia (17.1%) (Table 1).

The mean operative time was 30 ± 15 min, defined as skin-to-skin duration, and the mean intraoperative blood loss was 94.6 mL. The median length of hospital stay was 2.3 days (range: 1–7). All procedures were completed laparoscopically by two experienced bariatric surgeons. Adequate visualization of the angle of His and proximal stomach was achieved in all cases without requiring conversion to conventional liver retraction techniques, including in patients with severe obesity and hepatosteatosis.

Early postoperative complications occurred in 9.4% of patients. The most common complications included postoperative nausea and vomiting (3.8%), atelectasis (1.6%), wound infection (1.4%), and urinary tract infection (1.1%). Reoperation was required in three patients, including two cases due to leakage and one case due to postoperative bleeding. No mortality was observed, and the rate of intensive care unit admission was 0.2%. The 30-day readmission rate was 0.6% (Table 2).

According to the Clavien–Dindo classification, most postoperative complications were grade I–II events managed conservatively. The three patients requiring reoperation were classified as grade IIIb complications. No grade IV or grade V complications were observed.

**Table 2:** Perioperative and Postoperative Findings According to Clavien–Dindo Classification.

Parameters	Values	95% CI
Operative Findings		
-Operation time (minutes), Mean ± SD	30 ± 15	29.34–30.66
-Intraoperative blood loss (mL), Mean ± SD	94.6 ± 42.7	92.73–96.47
-Length of hospital stay (days), Median (min–max)	2.3 (1–7)	—
Conversion to open surgery, n (%) 0 (0)		
Additional trocar placement, n (%) 0 (0)		
Rescue liver retractor use, n (%) 0 (0)		
Early Complications (<30 days), n (%)	187 (9.4)	8.07–10.63%
Minor complications (Clavien–Dindo I–II), n (%)	184 (9.2)	
-Postoperative nausea/vomiting	76 (3.8)	
-Atelectasis	31 (1.6)	
-Surgical site infection	28 (1.4)	
-Urinary tract infection	22 (1.1)	
-Minor hemorrhage managed conservatively	4 (0.2)	
-Others 23 (1.15)	23 (1.15)	
Major complications (Clavien–Dindo IIIb), n (%) 3 (0.15)		0–0.32%
-Reoperation due to hemorrhage	1 (0.05)	
-Reoperation due to leakage	2 (0.10)	
Mortality, n (%)	0 (0)	
Intensive Care Unit Requirement, n (%)	4 (0.2)	
Readmission (<30 days), n (%)	12 (0.6)	0.26–0.94%

Notes: SD = Standard Deviation; 95% confidence intervals (95% CIs) were calculated for major perioperative outcomes using standard errors for continuous variables and binomial proportion methods for categorical variables.

**Figure 2:**
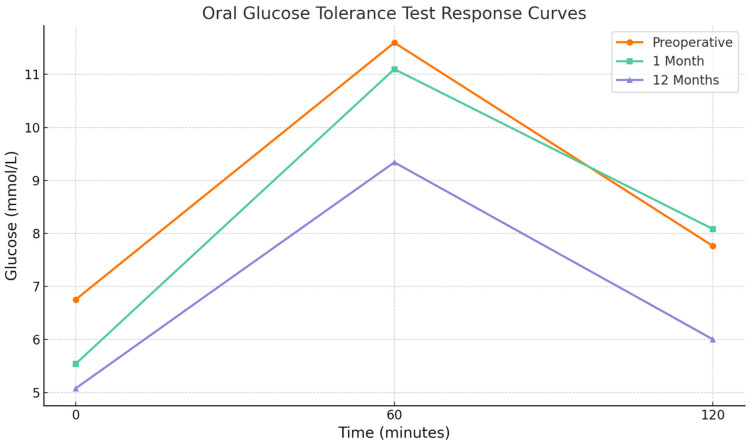
Postoperative changes in glycemic parameters during follow-up.

Significant improvements were observed in glycemic parameters following surgery. Mean HbA1c levels decreased from 7.23% preoperatively to 6.42% at 1 month and 5.67% at 12 months (*p* < 0.001). Similarly, HOMA-IR values decreased from 6.82 at baseline to 4.37 at 1 month and 2.84 at 12 months (*p* < 0.001). Fasting glucose levels declined from 148.3 mg/dL preoperatively to 126.4 mg/dL at 1 month and 98.7 mg/dL at 12 months (*p* < 0.001). A marked reduction in insulin and oral antidiabetic medication use was observed, and the overall diabetes remission rate at 12 months was 65.4% (Table 3, Figure 2).

**Table 3:** Changes in Glucose and Lipid Metabolism.

Parameters	Preoperative	95% CI	1st Month	95% CI	12th Month	95% CI	*p*-Value *
Glucose Metabolism Parameters							
HbA1c (%), Mean ± SD	7.23 ± 0.92	7.19–7.27	6.42 ± 0.84	6.38–6.46	5.67 ± 0.63	5.64–5.70	<0.001 ^abc^
HOMA-IR, Mean ± SD	6.82 ± 2.43	6.71–6.93	4.37 ± 1.92	4.29–4.45	2.84 ± 1.14	2.79–2.89	<0.001 ^abc^
Fasting glucose (mg/dL), Mean ± SD	148.3 ± 42.6	146.43–150.17	126.4 ± 28.7	125.14–127.66	98.7 ± 16.4	97.98–99.42	<0.001 ^abc^
Diabetes Status, n = 463							
Insulin users, n (%)	127 (27.4)	23.4–31.5	52 (11.2)	8.4–14.1	18 (3.9)	2.1–5.6	<0.001 ^abc^
Oral antidiabetic users, n (%)	336 (72.6)	68.5–76.6	248 (53.6)	49.0–58.1	142 (30.7)	26.5–34.9	<0.001 ^abc^
Remission ^†^, n (%)	-	–	163 (35.2)	30.9–39.6	303 (65.4)	61.1–69.8	<0.001 ^b^
Lipid Profile							
Total cholesterol (mg/dL), Mean ± SD	198.4 ± 42.7	196.53–200.27	182.3 ± 38.4	180.62–183.98	168.7 ± 32.6	167.27–170.13	<0.001 ^abc^
LDL (mg/dL), Mean ± SD	128.6 ± 36.4	127.00–130.20	112.4 ± 32.7	110.97–113.83	98.3 ± 28.4	97.06–99.54	<0.001 ^abc^
HDL (mg/dL), Mean ± SD	42.3 ± 8.7	41.92–42.68	46.8 ± 9.2	46.40–47.20	52.4 ± 10.1	51.96–52.84	<0.001 ^abc^
Triglycerides (mg/dL), Mean ± SD	186.4 ± 72.3	183.23–189.57	142.6 ± 54.8	140.20–145.00	108.7 ± 42.3	106.85–110.55	<0.001 ^abc^

Notes: * *p*-values: ^a^ Comparison between preoperative value and 1st month, ^b^ Comparison between preoperative value and 12th month, ^c^ Comparison between 1st month and 12th month. ^†^ Remission was defined as HbA1c < 6.5% without the use of antidiabetic medications. Statistical Analysis: Repeated measures ANOVA with post hoc Bonferroni tests were applied for continuous variables. Chi-square test was used for categorical variables. *p* < 0.05 was considered statistically significant; 95% confidence intervals (95% CIs) were calculated using standard errors for continuous variables and binomial proportion methods for categorical variables.

**Figure 3:**
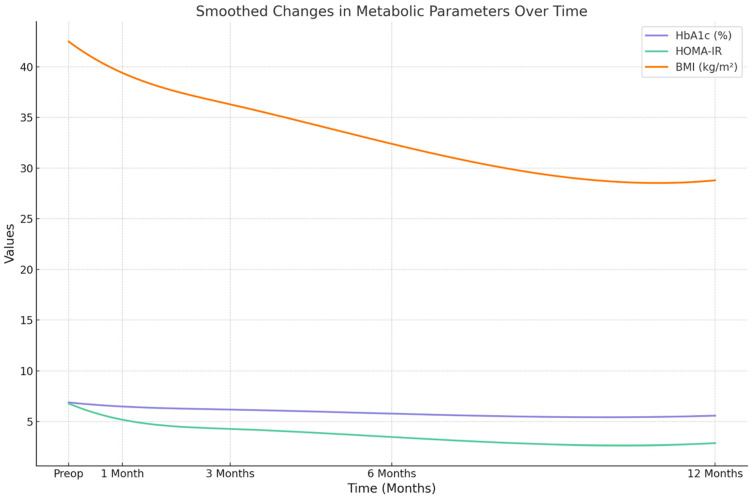
Changes in weight loss and metabolic parameters during postoperative follow-up.

A progressive and statistically significant reduction in BMI was observed over time, decreasing from 42.6 kg/m^2^ preoperatively to 36.3 kg/m^2^ at 3 months, 32.4 kg/m^2^ at 6 months, and 28.7 kg/m^2^ at 12 months (*p* < 0.001). The percentage of excess weight loss (%EWL) reached 42.3% at 3 months, 64.8% at 6 months, and 82.4% at 12 months, while the percentage of total weight loss (%TWL) reached 18.7%, 27.6%, and 34.2%, respectively (*p* < 0.001). Patients achieving diabetes remission demonstrated greater weight loss compared to those without remission, with significantly lower BMI values and higher %EWL and %TWL at 12 months (*p* < 0.05) (Table 4, Figure 3).

Subgroup evaluation of patients with BMI > 50 kg/m^2^ demonstrated that the three-port liver retractor-free technique remained technically feasible without the need for additional trocar placement or conversion to conventional liver retraction. Operative visualization and completion of sleeve gastrectomy were achieved laparoscopically in all patients within this subgroup.

**Table 4:** Weight Loss and Metabolic Outcomes.

Parameters	Preoperative	95% CI	3rd Month	95% CI	6th Month	95% CI	12th Month	95% CI	*p*-Value *
All Patients (n = 2000)									
BMI (kg/m^2^), Mean ± SD	42.6 ± 5.7	42.35–42.85	36.3 ± 4.8	36.09–36.51	32.4 ± 4.2	32.22–32.58	28.7 ± 3.9	28.53–28.87	<0.001 ^abcd^
%EWL, Mean ± SD	-		42.3 ± 12.4	41.76–42.84	64.8 ± 15.6	64.12–65.48	82.4 ± 18.2	81.60–83.20	<0.001 ^bcd^
%TWL, Mean ± SD	-		18.7 ± 5.3	18.47–18.93	27.6 ± 6.8	27.30–27.90	34.2 ± 7.4	33.88–34.52	<0.001 ^bcd^
Patients Achieving Diabetes Remission (n = 303) ^†^									
BMI (kg/m^2^), Mean ± SD	41.8 ± 5.4	41.19–42.41	35.2 ± 4.6	34.68–35.72	31.1 ± 4.0	30.65–31.55	27.4 ± 3.7	26.98–27.82	<0.001 ^abcd^
%EWL, Mean ± SD	-		44.7 ± 11.8	43.37–46.03	67.2 ± 14.9	65.52–68.88	85.6 ± 17.4	83.64–87.56	<0.001 ^bcd^
%TWL, Mean ± SD	-		19.4 ± 5.1	18.83–19.97	28.9 ± 6.5	28.17–29.63	35.8 ± 7.1	35.00–36.60	<0.001 ^bcd^
Patients Without Diabetes Remission (n = 160) ^†^									
BMI (kg/m^2^), Mean ± SD	43.2 ± 5.8	42.30–44.10	37.1 ± 4.9	36.34–37.86	33.4 ± 4.3	32.73–34.07	29.8 ± 4.0	29.18–30.42	<0.001 ^abcd^
%EWL, Mean ± SD	-		39.8 ± 12.7	37.83–41.77	61.4 ± 15.9	58.94–63.86	78.3 ± 18.6	75.42–81.18	<0.001 ^bcd^
%TWL, Mean ± SD	-		17.6 ± 5.4	16.76–18.44	25.8 ± 6.9	24.73–26.87	32.1 ± 7.6	30.92–33.28	<0.001 ^bcd^
Intergroup Comparisons ^‡^									
BMI	0.124		0.038		0.012		0.004		
%EWL	-		0.027		0.008		0.002		
%TWL	-		0.031		0.009		0.003		

Notes: * *p*-values: ^a^ Comparison between preoperative value and 3rd month, ^b^ Comparison between preoperative value and 6th month, ^c^ Comparison between preoperative value and 12th month, ^d^ Comparison between time points. Abbreviations: BMI = Body Mass Index; %EWL = Percent Excess Weight Loss; %TWL = Percent Total Weight Loss. ^†^ Of 463 patients with baseline DM2, 303 achieved remission, while 160 did not achieve remission. ^‡^ *p*-values for intergroup comparisons between patients with and without DM2 remission. The mean baseline BMI corresponded to Obesity Class III according to IFSO BMI-based classification; 95% confidence intervals (95% CIs) were calculated for all continuous variables using standard errors derived from the reported means, standard deviations, and sample sizes.

Histopathological examination revealed normal gastric mucosa in 19.6% of patients, while chronic gastritis was observed in 60.7% of cases. The coexistence of active and chronic gastritis was detected in 19.7% of patients. Helicobacter pylori positivity was identified in 26.2% of patients, with no significant differences according to age or gender. Intestinal metaplasia was detected in 1.6% of patients and was more frequent in individuals over 40 years of age, while atrophy was present in 0.9% of cases. Rare findings such as dysplasia, gastrointestinal stromal tumors, and neuroendocrine tumors were observed at very low rates and were not significantly associated with demographic variables (Table 5).

No statistically significant association was observed between Helicobacter pylori positivity and early postoperative complications, including leakage, postoperative bleeding, wound infection, or readmission within 30 days.

**Figure 4:**
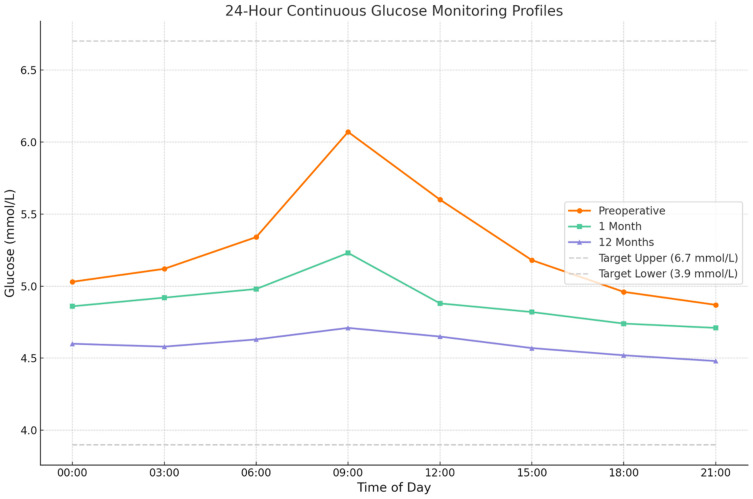
Representative 24-h continuous glucose monitoring profiles obtained during routine postoperative metabolic follow-up.

Analysis of 24-h continuous glucose monitoring (Medtronic, Northridge, CA, USA) demonstrated that preoperative glucose levels were consistently elevated, with peak values observed during midday. Postoperatively, glucose levels significantly decreased and remained largely within the target range, with further stabilization observed at 12 months. In addition, glycemic variability was markedly reduced following surgery, indicating improved glucose regulation (Figure 4).

The corresponding 95% confidence intervals for major perioperative and key metabolic outcomes are presented in Table 2, Table 3 and Table 4 and were consistent with the statistical significance observed in the primary analyses.

## 4. Discussion

In this large retrospective cohort study including 2000 consecutive individuals with obesity, we demonstrated that three-port laparoscopic sleeve gastrectomy (LSG) performed without the use of a liver retractor was feasible and associated with acceptable perioperative safety, favorable metabolic outcomes, and substantial weight loss during 12 months of follow-up. The findings of the present study suggest that elimination of the liver retractor can be technically achievable in selected bariatric surgery settings when performed by experienced surgeons.

Laparoscopic sleeve gastrectomy has become one of the most commonly performed metabolic bariatric surgery (MBS) procedures worldwide due to its reproducibility, relatively low morbidity, and significant metabolic benefits [7,8]. Consistent with previous studies, our results demonstrated marked improvements in glycemic control, insulin resistance, and lipid metabolism following LSG [13,14]. The observed reduction in HbA1c levels and the diabetes remission rate of 65.4% were comparable to previously reported outcomes in the literature. These metabolic improvements are likely related to the established hormonal and metabolic effects of sleeve gastrectomy itself, including altered incretin signaling, improved insulin sensitivity, and reduction in visceral adiposity [10,14,15].

From a technical perspective, the use of a liver retractor has traditionally been considered important for visualization of the proximal stomach and angle of His during LSG. However, liver retraction may also contribute to technical complexity and transient compression-related hepatic injury, particularly in individuals with obesity-associated hepatosteatosis [5]. In the present study, adequate exposure was achieved without the use of a mechanical liver retractor by optimizing patient positioning, controlled gastric traction, and abdominal wall manipulation. Importantly, no patient required additional trocar placement, conversion to open surgery, or rescue liver retraction during the procedures.

The overall complication rate of 9.4% observed in this study is comparable to rates reported in the literature for LSG [16]. Importantly, most postoperative complications were minor and managed conservatively according to the Clavien–Dindo classification system. Only three patients required reoperation, and no mortality was observed. These findings suggest that the three-port technique without liver retraction may be performed safely in experienced hands. However, because the present study did not include a control group undergoing conventional LSG, definitive conclusions regarding superiority or equivalence compared with standard approaches cannot be established.

The mean operative time of 30 min, calculated as skin-to-skin duration, was shorter than that reported in many published LSG series [16]. This finding may partially reflect procedural standardization, surgeon experience, and the high-volume nature of the study center. All procedures were performed by two experienced bariatric surgeons, which may have contributed substantially to operative efficiency and procedural consistency.

Minimally invasive surgery increasingly aims to reduce surgical trauma while maintaining procedural efficacy. Reduced-port and single-incision laparoscopic approaches have been associated with reduced postoperative pain, shorter recovery periods, and improved cosmetic outcomes [8,13]. The modified three-port technique evaluated in this study aligns with these minimally invasive principles by avoiding the need for an additional trocar dedicated to liver retraction. Nevertheless, postoperative pain scores, cosmetic satisfaction, and quality-of-life outcomes were not systematically assessed in the present cohort.

Substantial and sustained reductions in BMI were observed throughout follow-up, with %EWL reaching 82.4% and %TWL reaching 34.2% at 12 months. These findings are consistent with previous reports demonstrating effective weight reduction following LSG [17,18,19]. In addition, individuals achieving diabetes remission demonstrated greater weight loss compared with those without remission, suggesting a close relationship between metabolic improvement and postoperative weight reduction.

An additional important finding was the improvement in glycemic variability assessed by continuous glucose monitoring (CGM). Postoperative stabilization of glucose levels and reduction in glycemic fluctuations suggest enhanced metabolic regulation following LSG [20,21,22,23]. Because CGM data were retrospectively evaluated and monitoring protocols were not fully standardized, these findings should be interpreted descriptively.

Histopathological examination demonstrated a high prevalence of chronic gastritis and Helicobacter pylori positivity, findings that are consistent with previous bariatric surgery literature [24,25]. However, no statistically significant relationship was observed between Helicobacter pylori positivity and early postoperative complications in the present cohort. Although the clinical significance of preoperative Helicobacter pylori screening remains debated, eradication therapy may still be considered according to institutional protocols and regional prevalence patterns [26,27].

The present study also demonstrated that the liver retractor-free approach remained technically feasible in patients with severe obesity, including those with BMI >50 kg/m^2^. Adequate laparoscopic visualization was achieved without additional trocar placement or conversion in these patients, although objective visualization scoring systems were not applied.

### Limitations

This study has several limitations that should be considered when interpreting the findings. First, the retrospective design may introduce selection bias and limit the ability to establish causal relationships. Second, the absence of a control group undergoing conventional laparoscopic sleeve gastrectomy with liver retraction prevents direct comparison between techniques and limits conclusions regarding comparative safety or superiority. Third, the study was conducted at a single high-volume center, and all procedures were performed by two experienced bariatric surgeons, which may limit generalizability to lower-volume centers or less experienced surgical teams.

In addition, although follow-up was completed for all included patients through 12 months, longer-term follow-up is necessary to evaluate durability of weight loss, metabolic outcomes, and late complications. Postoperative pain, cosmetic satisfaction, quality-of-life outcomes, and liver perfusion parameters were not systematically evaluated. Furthermore, although continuous glucose monitoring findings were presented, CGM protocols were retrospectively derived from clinical practice rather than prospectively standardized. Finally, the absence of a comparator group prevents determination of whether the observed metabolic improvements were specifically related to the modified surgical technique rather than to sleeve gastrectomy itself.

Future prospective, randomized, multicenter comparative studies are warranted to further define the role, reproducibility, and potential advantages of liver retractor-free techniques in metabolic bariatric surgery.

## 5. Conclusions

Three-port laparoscopic sleeve gastrectomy performed without the use of a liver retractor appears to be a feasible and effective surgical approach in individuals with obesity when performed by experienced bariatric surgeons. In this large retrospective cohort, the technique was associated with acceptable perioperative safety, low rates of major complications, substantial weight loss, and significant improvements in metabolic parameters during 12 months of follow-up.

Adequate operative exposure was achieved without additional trocar placement, conversion to open surgery, or rescue liver retraction, including in patients with severe obesity. These findings suggest that a liver retractor-free approach may be technically achievable while maintaining minimally invasive surgical principles.

However, given the retrospective single-center design and the absence of a conventional comparison group, the present findings should be interpreted cautiously. The observed metabolic improvements cannot be attributed specifically to the modified surgical technique itself, as such outcomes are well-established effects of sleeve gastrectomy.

Further prospective, randomized, and multicenter comparative studies are needed to validate these findings and to better define the clinical role, reproducibility, and potential advantages of liver retractor-free techniques in metabolic bariatric surgery.

## Figures and Tables

**Table 1:** Demographic Data and Preoperative Characteristics.

Parameters	Values
Number of Patients (n)	2000
Age (years)	
- Mean ± SD	38.7 ± 9.3
- Median (min-max)	37.4 (22–65)
Gender, n (%)	
-Female	1423 (71.2)
-Male	577 (28.8)
BMI (kg/m^2^)	
-Mean ± SD	42.6 ± 5.7
-Median (min-max)	41.8 (35–55)
Associated Medical Problems, n (%)	
-Type 2 Diabetes Mellitus	463 (23.2)
-Hypertension	517 (25.9)
-Sleep Apnea	378 (18.9)
-Dyslipidemia	342 (17.1)
-Degenerative Joint Disease	283 (14.2)

Notes: BMI = Body Mass Index.

**Table 5:** Histopathological Findings.

Histopathological Findings	n (%)	Age ≤ 40 Years (n = 1248)	Age > 40 Years (n = 752)	Female (n = 1423)	Male (n = 577)	*p*-Value *
Histologically Normal Gastric Mucosa	392 (19.6)	248 (19.9)	144 (19.1)	-	-	0.842
Types of Gastritis						
Chronic Gastritis	1214 (60.7)	762 (61.1)	452 (60.1)	862 (60.6)	352 (61.0)	0.724 (age), 0.864 (sex)
-Minimal	187 (9.4)	116 (9.3)	71 (9.4)	-	-	0.893
-Mild	643 (32.2)	408 (32.7)	235 (31.3)	-	-	0.547
-Moderate	298 (14.9)	186 (14.9)	112 (14.9)	-	-	0.998
-Severe	86 (4.3)	52 (4.2)	34 (4.5)	-	-	0.867
Active + Chronic Gastritis	394 (19.7)	238 (19.1)	156 (20.7)	-	-	0.386
-Mild	198 (9.9)	122 (9.8)	76 (10.1)	-	-	0.762
-Moderate	164 (8.2)	98 (7.9)	66 (8.8)	-	-	0.524
-Severe	32 (1.6)	18 (1.4)	14 (1.9)	-	-	0.432
H. pylori Status						
Positive	523 (26.2)	318 (25.5)	205 (27.3)	368 (25.9)	155 (26.9)	0.382 (age), 0.642 (sex)
Negative	1477 (73.8)	930 (74.5)	547 (72.7)	-	-	-
Specific Findings						
Intestinal Metaplasia	31 (1.6)	12 (0.9)	19 (2.5)	22 (1.5)	9 (1.6)	0.008 (age), 0.927 (sex)
Atrophy	18 (0.9)	6 (0.5)	12 (1.6)	-	-	0.012
Dysplasia	4 (0.2)	1 (0.1)	3 (0.4)	-	-	0.124
GIST	2 (0.1)	0 (0)	2 (0.3)	-	-	0.167
Neuroendocrine Tumor	1 (0.05)	0 (0)	1 (0.1)	-	-	0.378

Notes: * *p*-values are for comparisons between age groups and gender groups. Chi-square test was applied for categorical variables; Fisher’s Exact test was used for cells with expected values < 5. *p* < 0.05 was considered statistically significant.

## Data Availability

The data that support the findings of this study are available from the corresponding author upon reasonable request.
